# Characterization of the GXXXG motif in the first transmembrane segment of Japanese encephalitis virus precursor membrane (prM) protein

**DOI:** 10.1186/1423-0127-17-39

**Published:** 2010-05-24

**Authors:** Ying-Ju Lin, Jia-Guan Peng, Suh-Chin Wu

**Affiliations:** 1 Department of Medical Research, China Medical University Hospital, Taichung 40402, Taiwan; 2 Institute of Biotechnology, Department of Life Science, National Tsing Hua University, Hsinchu 30013, Taiwan; 3 Vaccine Research and Development Center, National Health Research Institutes, Zhunan Town, Miaoli County, Taiwan

## Abstract

The interaction between prM and E proteins in flavivirus-infected cells is a major driving force for the assembly of flavivirus particles. We used site-directed mutagenesis to study the potential role of the transmembrane domains of the prM proteins of Japanese encephalitis virus (JEV) in prM-E heterodimerization as well as subviral particle formation. Alanine insertion scanning mutagenesis within the GXXXG motif in the first transmembrane segment of JEV prM protein affected the prM-E heterodimerization; its specificity was confirmed by replacing the two glycines of the GXXXG motif with alanine, leucine and valine. The GXXXG motif was found to be conserved in the JEV serocomplex viruses but not other flavivirus groups. These mutants with alanine inserted in the two prM transmembrane segments all impaired subviral particle formation in cell cultures. The prM transmembrane domains of JEV may play importation roles in prM-E heterodimerization and viral particle assembly.

## Background

Japanese encephalitis virus (JEV) is a small enveloped positive-strand RNA virus that belongs to the genus *Flavivirus *of the family *Flaviviridae *[1,2]. The RNA genome of all flaviviruses contain sequences that code for three structural protein genes (capsid C, membrane precursor prM, and envelope E) and seven non-structural protein genes (NS1, NS2A, NS2B, NS3, NS4A, NS4B, NS5), as well as flanking un-translated regions [1,2]. The flavivirus assembly process includes (i) interaction of prM and E proteins by heterodimer formation in the endoplasmic reticulum (ER), (ii) encapsulation of the genomic RNA by the C protein and enclosure by cell membrane-derived lipid bilayers containing prM and E proteins to form immature virions, and (iii) cleavage of the prM protein to M protein by furin or a furin-like protease in the trans-Golgi network (TGN) to release viral particles. Subviral particles (SPs) that do not contain genomic RNA and protein C have been found in flavivirus-infected cells [3]. Co-expression of prM and E envelope proteins resulted in the formation and secretion of SPs in cell cultures for tick-borne encephalitis virus (TBEV) [4], dengue virus (DENV) [5], JEV [6,7], Murray Valley encephalitis virus (MVEV) [8], St. Louis encephalitis virus (SLEV) [9], and West Nile virus (WNV) [10]. The interaction of prM and E proteins in flavivirus-infected cells is a major driving force of the assembly of virus although assembly mechanisms of the *Flaviviridae *are only very incompletely understood.

**Figure 1:**
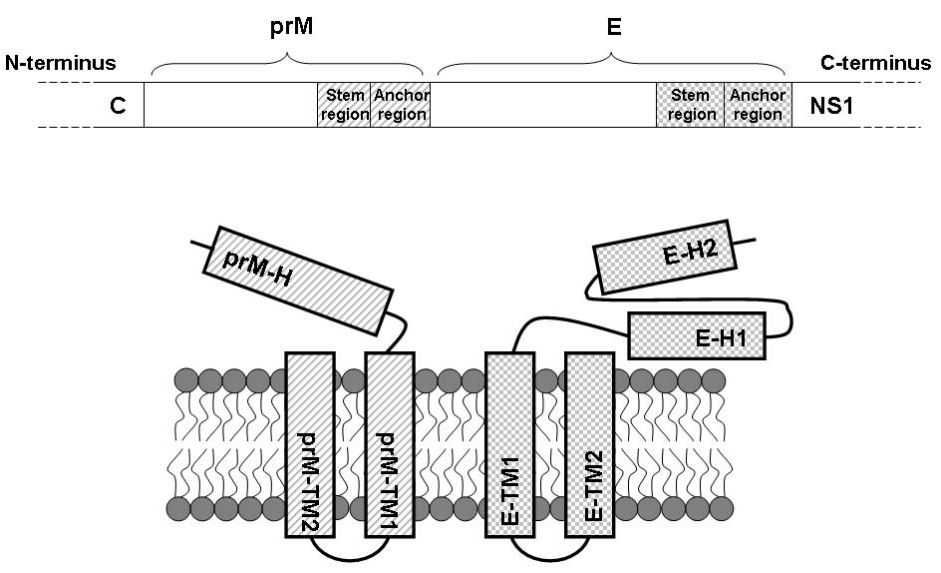
**Schematic dagram of flavivirus prM and E protein where the stem and anchor regions located at their C-terminal ends**.

The prM and E envelope proteins are type I transmembrane (TM) proteins and both contain stem and anchor regions at their C-terminal ends [11] as illustrated in Fig. 1 The stem region of the prM protein contains one helix domain (prM-H), and that of the E protein has two helix domains (E-H1, E-H2). The anchor regions of the prM and E proteins both contain two separate anchor domains (prM-TM1, prM-TM2, E-TM1, E-TM2). The stem and anchor regions of the prM and E proteins of DENV have both been predicted to include two alpha-helices [12]. High-resolution cryo-EM images of DENV show that the prM-H domain is partially buried in the outer lipid leaflet while the E-H1 and E-H2 domains are either angled or lie flat on the outer lipid leaflet [12]. The TM anchor regions of prM and E proteins (prM-TM1, prM-TM2, E-TM1, E-TM2) all form anti-parallel coiled-coil helices and do not penetrate the lipid membranes to come in contact with nucleocapsids [12]. In TBEV, the E-TM1 and E-H2 domains substantially influenced the stability of the prM-E interaction but did not affect the prM-mediated intracellular transport or secretion of soluble E protein according to C-terminal deletion analysis [13]. However, alanine insertion scanning mutagenesis in the anchor regions of prM and E of yellow fever virus (YFV) did not affect the prM-E interaction, but did inhibit SP formation [14]. Replacement of the stem and anchor regions of the E protein of DENV with that of JEV promoted SP production [15] but the E-H1 domain of JEV did not influence the SP secretion of DENV in CHO cells [16]. The E-TM2 domain of TBEV was demonstrated to be associated with virus particle formation, acting as a signal peptide for NS1 protein [17]. To our knowledge, most reported studies have focused on the stem and anchor regions of the E protein, and very little information is available for the prM protein.

In this work, the two anchor domains of prM protein (prM-TM1, prM-TM2) were studied using alanine insertion scanning mutagenesis to disrupt the helix-helix formation, and their roles in prM-E interaction and SP formation were characterized. Our earlier work has demonstrated that intracellular formation of the prM-E complex of JEV envelope proteins can be detected using baculovirus coexpression of prM and E *in trans*, and that the His-99 of prM significantly influences the prM-E interaction [18]. This paper reports that the GXXXG motif in the prM-TM1 domain influences the formation of prM-E heterodimers in infected cells. All of the mutants with alanine inserted in the prM-TM1 or prM-TM2 segment inhibited SP formation in culture supernatants, suggesting that the two TM segments of prM may play important roles for JEV particle assembly.

## Methods

### Cell lines and viruses

*Spodoptera frugiperda *Sf9 cells were grown at 28°C in TNM-FH medium (Sigma) supplemented with 10% fetal bovine serum (FBS). Hybridoma cells for production of the monoclonal antibodies (mAbs) 5B1 (anti-JEV prM) and E3.3 (anti-JEV E) were grown at 37°C in ISCOVE's modified Dulbecco's medium (Invitrogen) supplemented with 10% FBS. The JEV strain CH2195LA [GenBank:AF221499] [19,20] and recombinant baculoviruses were produced and propagated in Sf9 cells.

### Construction of recombinant baculoviruses

Plasmid pUC18-prME containing nucleotides 414 to 2477 from JEV virus strain CH2195LA was the parent construct used for alanine-insertion mutants, substitution mutants and wild type controls. Alanine-insertion mutants at appropriate positions of prM were constructed using overlapping PCR. Substitution mutants at position 142 and 146 of prM were also constructed using overlapping PCR. All constructs contained *BamHI *and *EcoRI *restriction sites for ligation to the pBlueBac4 baculovirus vector (Invitrogen). The constructed recombinant pBlueBac4 plasmids were co-transfected with a linearized baculovirus Bac-N-Blue DNA (Invitrogen) to Sf9 cells treated with Cellfectin (Invitrogen) and incubated for 5-7 days. Recombinant baculoviruses were obtained from the blue-stained plaques formed in the infected Sf9 cells overlaid with agarose medium that contained 5-bromo-4-chloro-3-indoyl-β-D-galactoside (X-gal). Three plaque purifications were performed to obtain the recombinant baculoviruses that were used in this study.

### Western blotting

Sf9 cells co-infected with recombinant baculoviruses were harvested and analyzed with 12% SDS-PAGE under reducing condition. Infected cell lysates were electrically transferred onto the nitro-cellulose membranes, blocked by 5% skim milk, and reacted with mAbs 5B1 and E3.3, respectively. As an internal control, the glyceraldehyde-3-phosphate dehydrogenase (GAPDH) was parallel used and detected by anti-GAPDH mAb (SignalChem). All of these samples were then reacted with anti-mouse IgG conjugated to peroxidase (KPL) and visualized using ECL (PerkinElmer).

### RNA extraction and complementary DNA preparation by RT

The co-infected Sf9 cells at 3 days post infection were harvested and immediately extracted for total RNAs using RNeasy mini kit (Qiagen) according to the protocol of the supplier, and subsequently quantified with a Nano-Drop ND-1000 spectrophotometer (NanoDrop Technologies). RNA purity was estimated by the absorbance ratio A_260_/A_280_. The calculated ratios were in the range of 1.8 to 1.9 from the indicated samples and indicate high purity RNAs. One microgram total RNA was reverse transcribed using the M-MLV Reverse Transcriptase (Promega) with 250 ng random hexamer primers (Invitrogen) according to the manufacturer's instructions, without an RNase inhibitor in a final volume of 20 μL. The mixture was incubated for one hour at 37°C.

### Quantitative real-time PCR

Quantitative real-time PCR was performed in white LightCycler 480 Multiwell Plate 96 plates (Roche) in a final reaction volume of 10 μL. For detection of prM mRNA transcripts [GenBank:AF221499]: Forward primer 5'- aggaatcctggctacgcttt -3', reverse primer 5'-cgttgttactgccaagcatc -3' and specific probe 5'-ggcggcgg -3'. For detection of E mRNA transcripts [GenBank:AF221499]: Forward primer 5'- gaaggggagcattgacacat -3', reverse primer 5'-gattgttctcccaatcgcttt-3' and specific probe 5'-ttctcctg -3'. For detection of GAPDH mRNA transcripts [GenBank:NM_080369]: Forward primer 5'-aagggaatcctgggctacac-3', reverse primer 5'-aatgggtgtcgctgaagaag-3' and specific probe 5'-ggaggtgg -3'. According to the primer optimization matrix, varying amounts of the forward and reverse primer (Invitrogen) of 100 nmol/L, 200 nmol/L, and 300 nmol/L were mixed in 1× LightCycler 480 Probes Master (Roche) containing 100 nmol/L and 200 nmol/L of the human Universal Probe Library probe (Roche), respectively, and 1.0 μL of cDNA as template (1:20, taken from one appropriate source described above, but consistent throughout a single experiment). The initial denaturation (95°C, 10 minutes) was followed by 45 cycles of 10 seconds at 95°C, 30 seconds at 60°C, and a final cooling step at 40°C for 10 seconds. Each primer concentration combination was analyzed in duplicate for each cDNA source used.

### PNGaseF and EndoH glycosidase treatment of glycoprotein

Sf9 cells infected by recombinant baculoviruses were harvested and lysed using a lysis buffer for 3 hours at 4°C. Then, 10X glycoprotein denaturing buffer (New England BioLab) was added to the cell lysate to a 1X concentration and the whole was boiled at 95°C for 5 minutes. Subsequently, 14 μL of denatured sample was mixed with 2 μL 10X G7 buffer, 2 μL 10% NP-40, and 2 μL PNGaseF or EndoH to yield a 20 μL reaction mixture. Finally, the reaction mixtures were left to stand at 37°C for 3 hours to allow deglycosylation.

### Confocal laser scanning microscopic analysis for subcellular localization

Infected Sf9 cells that had been grown on 15 mm glass coverslips for 24 hour post infection (hpi) were washed three times in TBS (140 mM NaCl; 25 mM Tris-HCl, pH 6.1) incubated in a medium that contained 10 nM DiIC13(3) (an ER-staining dye, Molecular Probes) for 30 min, and finally fixed with 3.7% paraformaldehyde for 1 h. The cells were then permeabilized for 1 h with TBS containing 0.1% Triton X-100. The expression of JEV proteins in these cells was revealed by staining with the anti-E mAb E3.3 or anti-prM mAb 5B1 followed by goat anti-mouse Alexa Fluor 647 IgG (Molecular Probes). The subcellular localizations in these cells were examined by confocal laser scanning microscopy (Zeiss LSM 500) using a 100x 1.3 oil objective.

### Metabolic labeling and sucrose gradient sedimentation analysis

Subconfluent monolayers of Sf9 cells grown in 6-well plates were infected with recombinant baculoviruses at multiplicity of infection (MOI) = 5. At 24 hpi, the TNM-FH medium was removed and replaced with TNM-FH medium that contained 0.01% normal methionine (Met) and 0.02-0.10 mCi/mL of [^35^S]methionine/cysteine. Baculovirus-infected Sf9 cells were treated with a lysis buffer (50 mM Tris-HCl [pH 8.0], 150 mM NaCl, 2 mM EDTA, 1% Triton X-100 and 1 mM phenylmethylsufonylfluoride), and then pre-cleared using protein A Sepharose (Pharmacia). Each purified cell lysate was layered on a 3-60% (wt/wt) sucrose gradient in gradient buffer (50 mM Tris-HCl [pH 8.0], 150 mM NaCl, 2 mM EDTA, 0.5% Triton X-100, 1 mM phenylmethylsufonylfluoride). The gradients were centrifuged in a Hitachi RPS40ST rotor at 38,000 rpm and 15°C for 22 h. After centrifugation, each fraction was collected, immunoprecipitated using mAb E3.3 (anti-JEV E protein) and incubated for 5 h at 4°C; then protein A Sepharose slurry was added and the system was incubated for another 16 h under the same conditions. The immunoprecipitates were separated by centrifugation at 2500 rpm for 3 min and the pellets thus formed were washed twice with incubation buffer (63 mM Tris-HCl [pH 6.8]). The precipitated material was solubilized by heating (95°C for 5 min) with a reducing-electrophoresis sample buffer (50 mM Tris-HCl pH6.8, 2% SDS, 0.1% bromophenol blue, 5% 2-mercatoethanol, and 10% glycerol) and further analyzed by SDS-PAGE (12%) and fluorography. At least two independent experiments were performed to obtain the reproducible results.

### Enzyme-Linked Immunosorbent Assay (ELISA) for quantification of E protein

Samples from each fraction were diluted in coating buffer (0.1 M Na_2_CO_3 _[pH 9.6]) and used to coat 96-well microplates (Corning, Costar 9018) by overnight incubation at 4°C. Following incubation, the samples were blocked using 3% bovine serum albumin (BSA) for 2 h at room temperature, and the solid phase was then reacted with mAb E3.3 (anti-E monoclonal antibody; 1:500 dilution) for 2 h at room temperature. The bound antibodies were detected following incubation with the anti-mouse IgG conjugated to peroxidase (KPL; 1:2000 dilution) for 1 h at room temperature. The ELISA products were developed using a chromogen solution containing 2, 2'-azino-di-(3-ethylbenzthiazoline-6-sulfonate; ABTS) and hydrogen peroxide and their absorbance values were measured at 405 nm. The levels of E proteins were calculated from the absorbance values by comparison with a standard curve established with purified domain III, which also reacted with mAb E3.3 at a known protein concentration [18]. The ELISA data were further normalized with the relative total E protein expression level, which was achieved by treating the baculovirus-infected Sf9 cells with 10 μg/ml Brefeldin A (Merck), a fungal macrolide antibiotic which disassembly the golgi compartment and blocks the secretion. The relative total E protein expression level was determined by the intensity of Western blotting bands with software Gel-Pro Analyzer (Media Cybernetics).

### Transmission electronic microscopy with immuno-gold labeling

The culture supernatants of the baculovirus-infected Sf9 cells (MOI = 2.5) or JEV-infected Sf9 cells at 5 dpi were centrifuged, precipitated using 10% polyethylene glycol (PEG)- 8,000, and then resuspended in 1 ml TN buffer (10 mM Tris-HCl pH7.5, 100 mM NaCl). The suspensions were subjected to centrifugation in a continuous sucrose gradient (3-60% wt/wt) at 38,000 rpm for 24 h at 4°C (RPS40ST rotor, HITACHI 85P-72 ultracentrifuge). The virus particles were collected from the fractions in 13 tubes with volumes of 0.85 mL for transmission electron microscopy (TEM). A single-droplet negative staining procedure was used for TEM visualization. Sample droplets of 30 μL were absorbed for 10 min onto Formvar carbon-coated copper grids (200 mesh; Agar scientific), washed in distilled water, and then stained with 2% uranyl acetate for 1 min. After drying in air, the negatively-stained samples were labeled with anti-prM mAb 5B1 or anti-E mAb E3.3 for 1 h, and then with anti-mouse IgG conjugated with 5 nm gold nanoparticles for 1 hr. The prepared grids were examined in a HITACHI H-7500 TEM at 100 kV.

## Results

### Expression of prM proteins with inserted alanine in co-infected Sf9 cells

**Figure 2:**
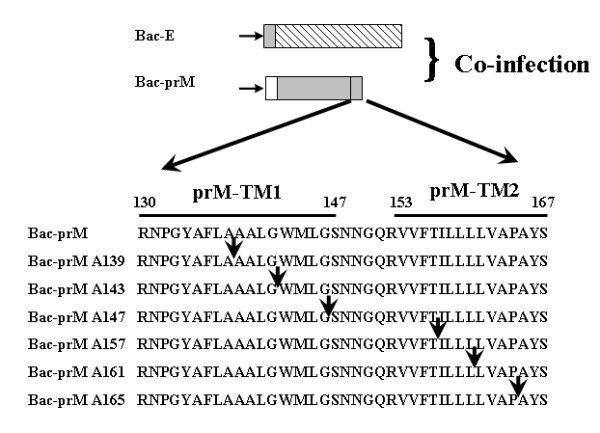
**Positions of the alanine insertion mutations in the two transmembrane regions (TM1 and TM2) of prM protein of JEV CH2195LA strain**. A series of mutants was constructed; Bac-prM, Bac-prM A139, Bac-prM A143, Bac-prM A147, Bac-prM A157, Bac-prM A161 and Bac-prM A165. Arrows indicate the positions of alanine insertion.

The prM anchor region of the JEV 2195 strain contains the prM-TM1 (residues prM_131-147_) and prM-TM2 (residues prM_153-167_) segments [7] (Fig. 2); these two segments can form helix-helix complexes in lipid bilayers. Since alanine insertion scanning mutagenesis is typically used to identify the residues critical for helix-helix interactions [21,22], we constructed a series of prM mutants with alanine inserted in the prM-TM1 and prM-TM2 segments including prM139, prM143, prM147, prM157, prM161, and prM165 (Fig. 2). The positions of alanine insertion in the prM-TM1 and prM-TM2 segments were adopted from a previous study on helix-helix interactions in the anchor region of the YFV prM protein [14]. In this study, we investigated the potential role of the TM domains of the JEV prM protein in prM-E heterodimerization as well as in SP formation. The analyses were conducted using a baculovirus expression system with prM and E expressed *in trans*. The signal peptide of E protein is located at the C-terminal end of prM protein, approximately in the prM-TM2 region. Use of the prM and E coexpression *in trans *provides the advantage to study the prM TM domains without affecting the signal peptide of E protein for E protein expression, translocation, and the interactions with prM as a heterodimeric complex formation as we reported previously [7].

**Figure 3:**
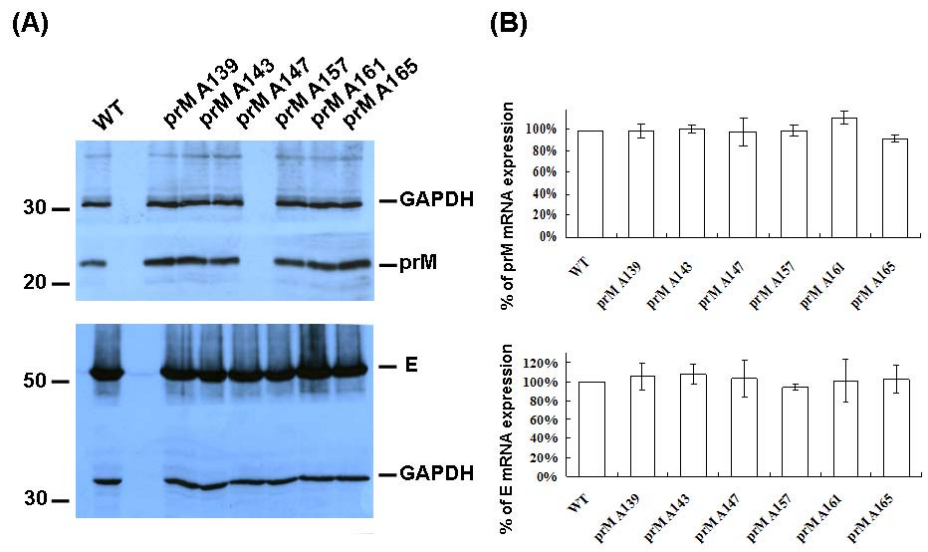
**(A) The total expression of prM and E proteins in Sf9 cells co-infected with Bac-prM (wild type and mutants A139, A143, A147, A157, A161, A165) and Bac-E**. GAPDH was measured as an internal control; (B) The mRNA transcript levels of prM and E in the co-infected Sf9 cells measured by real-time RT-qPCR. The wt prM/E expression level was taken as 100%.

**Figure 4:**
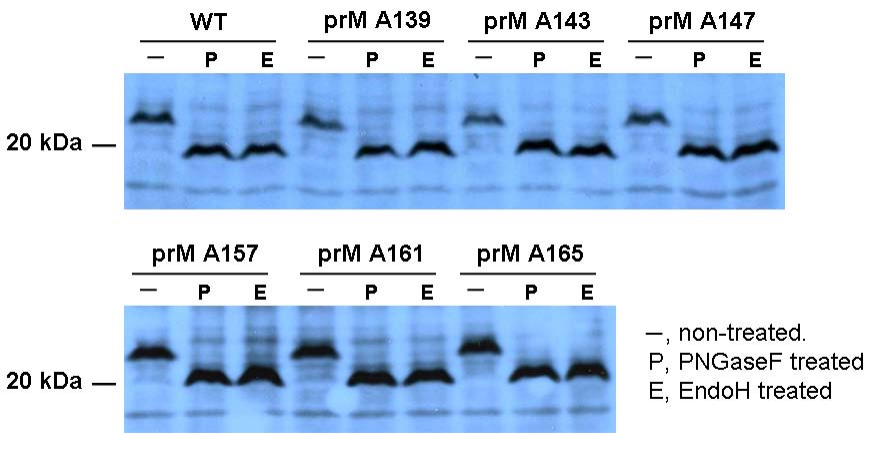
**Treatment of recombinant prM with endoglycosidase PNGaseF and EndoH**. The prM mutants with inserted alanine were treated with PNGaseF (P) and EndoH (E) to analyze the presence and composition of their N-linked glycans.

**Figure 5:**
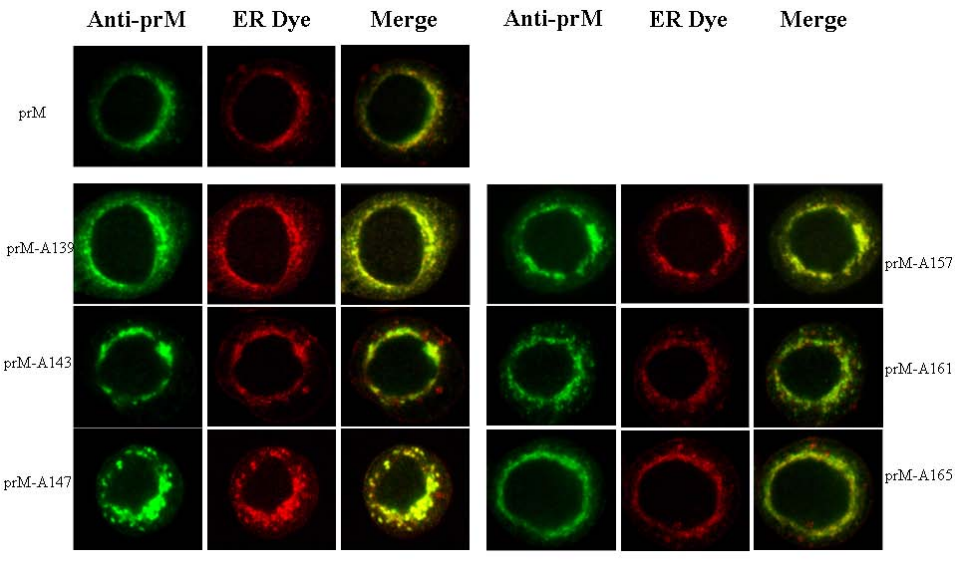
**Observation of subcellular localization using confocal laser scanning microscopy**. The subcellular location of wild type and mutant prM was visualized by double-staining of the infected Sf9 cells. The ER compartment was stained with 10 nM DiIC13(3), and the prM proteins were stained with mAb 5B1, followed by goat anti-mouse Alexa Fluor 647 IgG. Photographs were taken with appropriate excitation laser wavelengths and merged to reveal the co-localization of prM and ER compartment.

To demonstrate that the prM proteins with inserted alanine did not influence the expression or subcellular localization of the prM-E interaction, Sf9 cells were co-infected with Bac-prM (wild-type and mutants) and Bac-E at MOI = 5. These alanine-inserted mutations in the prM-TM1 and prM-TM2 segments did not affect the total expression of E and prM proteins in the co-infected Sf9 cells (Fig. 3A). The mRNA transcript levels of prM and E of the prM mutants in the co-infected Sf9 cells were similar as measured by using RT-quantitative real time PCR methods (Fig. 3B). Furthermore, the cell lysates of the co-infected Sf9 cells treated with PNGaseF or EndoH enzymes did not show differences in their glycoprotein patterns in SDS-PAGE gels, suggesting these alanine-inserted prM mutants also did not affect the intracellular localization of mutant prM proteins (Fig. 4). The intracellular localization of the alanine-inserted prM proteins was further confirmed using confocal laser scanning microscopy. Sf9 cells that were infected with Bac-prM (wild type) or Bac-prM (alanine-insertion mutants) were doubly stained with a prM-specific MAb and DiC, an ER-specific dye. The doubly stained images obtained by confocal laser scanning microscopy indicated that the intracellular distributions of prM and the prM proteins with inserted alanine almost overlapped with the ER marker (Fig. 5). Thus, none of the prM mutants with inserted alanine influenced the expression or subcellular localization of the prM protein in the co-infected Sf9 cells.

### Effects of prM proteins with inserted alanine on prM-E heterodimerization in co-infected Sf9 cells

**Figure 6:**
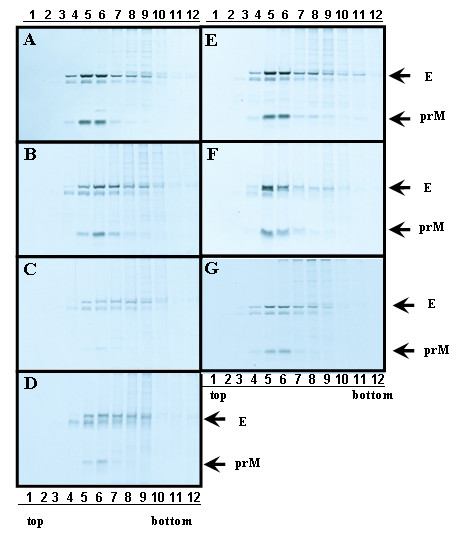
**Sucrose gradient sedimentation analysis of alanine-insertion mutagenesis of the anchor region of prM protein**. Sf9 cells were coinfected with Bac-E and Bac-prM mutants. Cell lysates labeled with [^35^S] were applied for centrifugation in a 3 to 60% (wt/wt) sucrose gradient. Each fraction was immunoprecipitated with monoclonal antibody E3.3. The immune complexes were analyzed by reducing SDS-PAGE and fluorography. (A) Sf9 cells coinfected with Bac-prM and Bac-E; (B) Sf9 cells coinfected with prM A139 and Bac-E; (C) Sf9 cells coinfected with prM A143 and Bac-E; (D) Sf9 cells coinfected with prM A147 and Bac-E; (E) Sf9 cells coinfected with prM A157 and Bac-E; (E) Sf9 cells coinfected with prM A157 and Bac-E; (F) Sf9 cells coinfected with prM A161 and Bac-E; (G) Sf9 cells coinfected with prM A165 and Bac-E. The data presented in this figure are three independent experiments.

**Figure 7:**
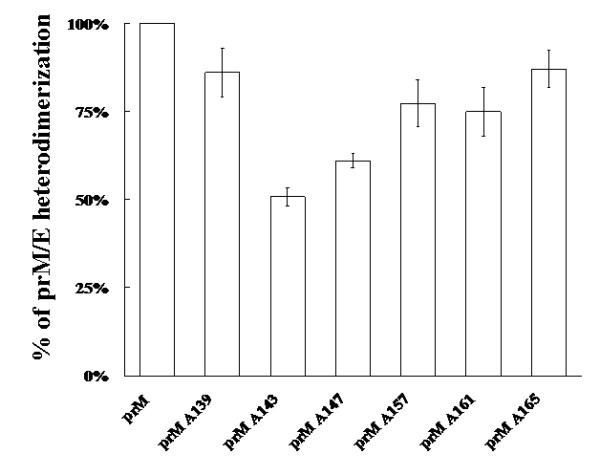
**prM-E heterodimerization of prM mutants with inserted alanine as a percentage of the binding intensity of wild type prM**. Each prM to E ratio was calculated. The wild type prM/E ratio was taken as 100% in evaluating the mutant prM-E binding affinity. Each data represents the average of the experiments for a single prM mutant and the error is standard deviation. The positions of the alanine insertions in the TM region of prM are indicated.

To investigate the alanine-inserted prM mutants affecting prM-E heterodmerization, Sf9 cells co-infected with Bac-prM (wild type or alanine-insertion mutants) and Bac-E were radiolabelled with [^35^S]methionine-cysteine medium for 24 h, purified using sucrose gradient sedimentation, and immunoprecipitated by an E-specific mAb. The results in SDS-PAGE gels showed that the two reactive bands (corresponding to prM of 21 kDa and E of 56 kDa) were present in sucrose gradient fractions 5 and 6 of the co-infected Sf9 cells with Bac-prM (wild type) (Fig. 6A), Bac-prM A139 (Fig. 6B), Bac-prM A143 (Fig. 6C), Bac-prM A147 (Fig. 6D), Bac-prM A157 (Fig. 6E), Bac-prM A161 (Fig. 6F) and Bac-prM A165 (Fig. 6G). However, the intensity of binding to the prM-E complex by some prM mutants with inserted alanine differed from the wild type prM. For instance, a smaller amount of E protein was precipitated when coexpressed with prM A143, prM A147, or prM A165, compared to those of E protein when coexpressed with the WT or other mutant prM proteins. To characterize these mutations in the prM-TM1 and prM-TM2 that affected the association between E and prM, the percentage of heterodimerization of prM to E was taken as the prM/E ratio. The prM/E ratio was measured for the stable prM-E complex in each sucrose gradient fractionation to minimize the differential precipitation efficiency in each sample. Also a side-by-side comparison of the total levels of E and prM prior to precipitation was also conducted where we did not find differences of prM and E expression for the WT and mutants (data not shown). The prM/E ratios in the sucrose gradient fractions 4 to 6 were 100% (Bac-prM), 86% (Bac-prM A139), 51% (Bac-prM A143), 61% (Bac-prM A147), 77% (Bac-prM A157), 75% (Bac-prM A161), and 87% (Bac-prM A165) (Fig. 7). The Bac-prM A143 mutant most reduced the formation of prM-E heterodimers in infected cells.

### Effects of prM proteins with inserted alanine on SP formation in co-infected Sf9 cells

**Figure 8:**
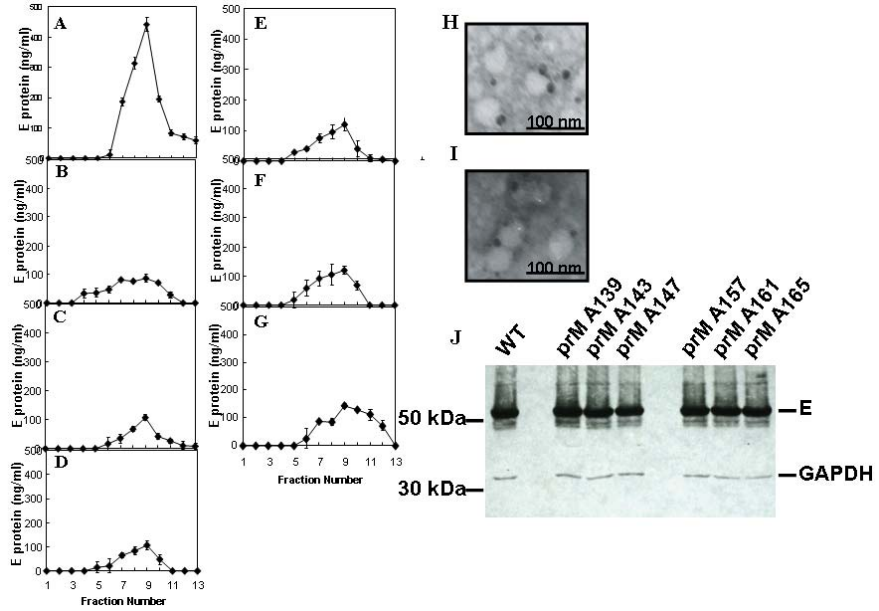
**Alanine-insertion mutagenesis of the TM regions of prM protein: effect on SP formation**. Recombinant SPs secreted from Sf9 cells co-infected with with Bac-E and (A) Bac-prM; (B) Bac-prM A139; (C) Bac-prM A143; (D) Bac-prM A147; (E) Bac-prM A157; (F) Bac-prM A161, and (G) Bac-prM A165 were quantified by ELISA. The size and shape of the secreted recombinant SP were observed by TEM using immuno-gold labeling with (H) mAb E3.3 and (I) and mAb 5B1. (J) The total E protein in Sf9 cells was determined in the presence of Brefeldin A, a fungal a fungal macrolide antibiotic which blocks E protein secretion. GAPDH was taken as an internal loading control.

**Figure 9:**
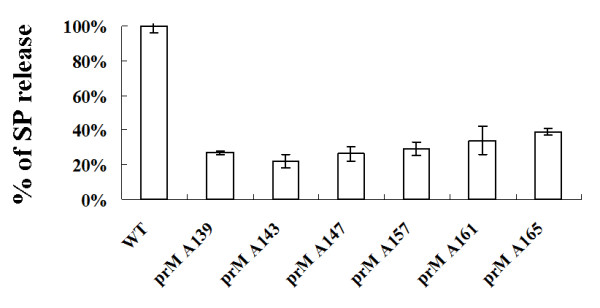
**Quantification of the SP release was determined by calculating the underneath area of the E protein concentrations from fractions 7 to 10 in Fig. 7 A-G**. The underneath areas were normalized by the corresponding total amount of E protein in Fig 7J and the percentage of SP release represented the relative value of each prM mutant (Fig. 7B-G) compared to the wild type (Fig. 7A)

To further evaluate the SP formation affected by the alanine-inserted prM mutants, culture supernatants were collected from Sf9 cells that were co-infected with Bac-prM (wild type or alanine-insertion mutants) and Bac-E, purified using sucrose gradient sedimentation, and the E protein concentration in each sucrose fraction was measured by ELISA. The results showed that much less SP was formed in Sf9 cells that were infected with any of the prM mutants with inserted alanine than wild type Bac-prM (Fig. 8A-G). The release of SP from Sf9 cells that were co-infected with the wild-type Bac-prM (Fig. 8A) was verified by TEM using immuno-gold labeling with the anti-E mAb E3.3 (Fig. 8H) and the anti-prM mAb 5B1 (Fig. 8I). Quantification of the SP release from the co-infected Sf9 cells was determined by calculating the underneath area of the E protein concentrations. The results were further normalized to the total E protein expression of the same co-infected cells treated with brefeldin A to prevent SP release (Fig. 8J). Thus, the percentage of SP release in the co-infected cells was calculated from the underneath area of sucrose gradient fractions of culture supernatants of the co-infected Sf9 cells and the value was normalized to the total E protein contents measured in brefeldin A-treated Sf9 cells. The results showed the percentage of SP release dropped from 100% (Bac-prM) to 27% (Bac-prM A139), 22% (Bac-prM A143), 26% (Bac-prM A147), 29% (Bac-prM A157), 34% (Bac-prM A161), and 39% (Bac-prM A165) (Fig. 9). All the prM mutants with inserted alanine resulted in significant impairment for the formation of SP in baculovirus-infected Sf9 cells, suggesting that the two TM domains of prM may play important roles for the formation and release of SP.

### Glycine substitution mutagenesis in the prM-TM1 segment

**Figure 10:**
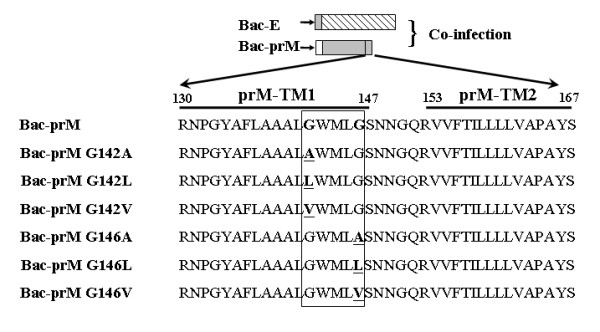
**Characterization of the GXXXG motif of the TM1 region of the prM protein by glycine-substitution mutagenesis**. Glycine residues at 142 and 146 were substituted by alanine, leucine, or valine to investigate the relationship between the GXXXG motif and the prM-E binding affinity.

**Figure 11:**
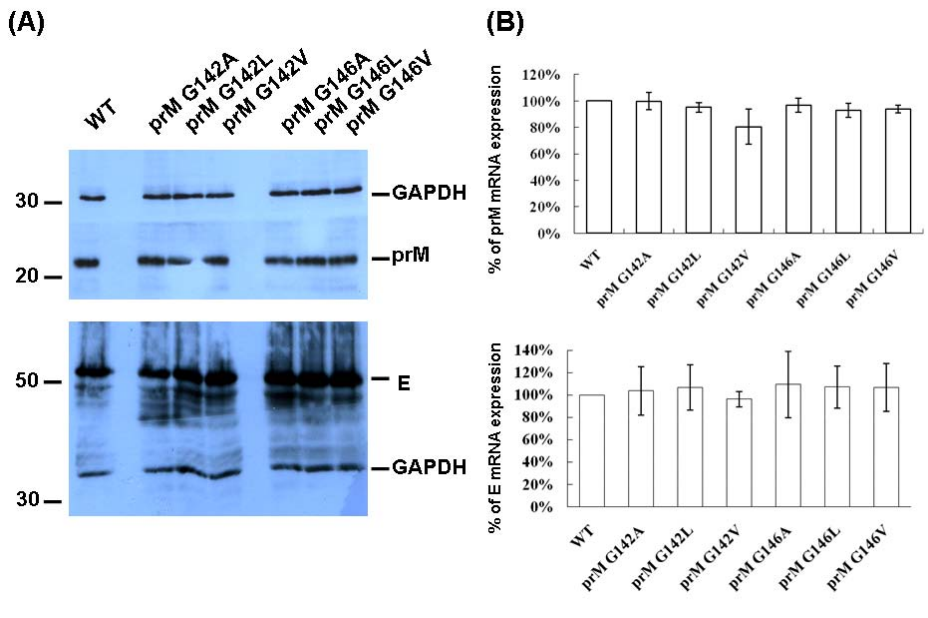
**(A) The total expression of prM and E proteins in Sf9 cells co-infected with Bac-prM (wild type and mutants G142A, G142L, G142V, G146A, G146L, G146V) and Bac-E**. GAPDH was measured as an internal control; (B) The mRNA transcript levels of prM and E in the co-infected Sf9 cells measured by real-time RT-qPCR. The wt prM/E expression level was taken as 100%.

**Figure 12:**
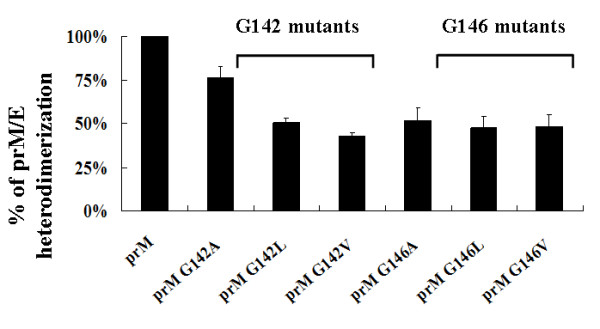
**The percentage of the binding intensity of wild type prM**. Each prM to E ratio was calculated where wild type prM/E ratio was taken as 100% in evaluating the glycine-substituted mutant prM-E binding affinity. Each data represents the average of the experiments for a single prM mutant and the error is standard deviation.

To further characterize the prM A143 mutant that influenced the prM-E interaction, the prM-TM1 sequences were analyzed and a GXXXG motif was identified at residues 142-146 (G_142_WMLG_146_S_147_) (see Fig. 1). The GXXXG motif contains amino acids that are hydrophobic and commonly present in many TM proteins [23]. Therefore the glycine at residues 142 and/or 146 were replaced with alanine, leucine or valine, respectively, to demonstrate the specificity of the GXXXG motif (Bac-prM G142A, Bac-prM G142L, Bac-prM G142V, Bac-prM G146A, Bac-prM G146L, Bac-prM G146V) (Fig. 10). Similarly, these GXXXG mutants did not affect the total expression of E and prM proteins in the co-infected Sf9 cells (Fig. 11A). The mRNA transcript levels of prM and E of these GXXXG mutants in the co-infected Sf9 cells were around the same as measured by using RT-quantitative real time PCR methods (Fig. 11B). However, the percentage of heterodimerization of prM to E for characterization of the mutations that affected the association between E and prM revealed the glycine substitutes at 142 and 146 by alanine, leucine or valine reduced by approximately 50%, except the mutant G142A giving a 25% loss (Fig. 12). Therefore, both glycine residues in the GXXXG-motif of the prM-TM1 segment are equally important to the formation of a stable prM-E complex.

### Sequence alignment analysis of TM regions of prM proteins of different flavivirus groups

The amino acid sequence alignments of the prM TM regions of 5 groups of flaviviruses, including JEV sero-complex viruses (JEV, KUNV, MVEV, SLEV, WNV), four-serotype dengue viruses (DENV-1, DENV-2, DENV-3, DENV-4), TBEV, and YFV were analyzed to determine whether the GXXXG motif was conserved. These sequences were aligned and analyzed using the Showalign program from Jemboss on the Jemboss server http://bioinfo.life.nthu.edu.tw/bioinfo.htm. Additional file 1 summarizes the results, which show that the amino acid sequences of the prM-TM1 are nearly identical except amino acid 140 in three strains of JEV but vary among other JEV serocomplex viruses, DENV-1, DENV-2, DENV-3, DENV-4, TBEV, and YFV. However, the GXXXG motif is present in all JEV serocomplex viruses.

## Discussion

The flavivirus assembly process is driven mainly by the interactions between two envelope proteins prM and E. Co-expression of these two proteins in cultured cells can yield recombinant SPs. In this work, alanine insertion and site-directed mutagenesis were employed to study the influence of the TM anchor region of prM protein on the formation of prM-E heterodimers and SP production. The results show that the GXXXG motif present in the prM-TM1 domain is the element that affects the formation of prM-E heterodimers. Both TM segments of the prM protein are crucial for SP formation and release.

Alanine-insertion mutagenesis was employed to disrupt the TM helical structures in the TM anchor regions of prM protein (prM-TM1 and prM-TM2) in experiments. Six prM mutants with inserted alanine (prM139A, prM143A, prM147A, prM157A, prM161A, prM165A) were used; the insertion sites were chosen according to a study of alanine insertion mutagenesis of the TM proteins of YFV [14]. This insertion mutagenesis has been applied for identifying critical segments of TM region involved in helix-helix interactions [21,22,24]. These mutated proteins may also have subtle alternations in protein stability, expression, folding, and translocation. Baculovirus insect cell expression has been widely used to express complex proteins particularly requiring post-translational modifications [25]. In this study we employed a baculovirus coexpression system with prM and E expressed *in trans *where alanine insertion and substitution mutagenesis of prM-TM1 and prM-TM2 fragments were conducted to study the interactions of prM and E proteins in co-infected Sf9 cells. Our results show that a series of alaine inserted prM mutants neither affected the mRNA and protein expression levels of prM nor the subcellular localization of the prM protein in Sf9 cells. However, whether these prM mutants may alter anchorage of mutant prM proteins into membranes or cause a conformational change of the extracellular domain of prM are still required for further confirmation. Among these alanine-inserted prM mutants, the prM-143A mutant within the GXXXG motif in the prM-TM1 segment markedly reduced the formation of the prM-E heterodimers by up to 50%. This result is consistent with our earlier study using C-terminal truncation, which showed a reduction of the formation of the prM-E complex by approximately 40% for the TM1 of prM protein, as compared to the complete abolishment of the interactions by the prM His-99 mutation [7].

The impairment of SP formation but a modest disruption of prM-E heterodimerization by all prM mutants with inserted alanine was observed in our study. For example, the mutant prM165 exhibited an interaction profile nearly identical to wild type in the prM-E heterodimerization (Fig. 7), yet had a significant effect on SP release (Fig. 9). Conversely, mutants with a more "dramatic" reduction in the interaction between prM and E displayed similar reductions in the release of SP. Mutations in the two prM TM segments may increase the heterogeneity of SP, as evidenced by the broader peak of E protein in these mutants (Fig. 8B-G). The broaden fractions of SPs from prM mutants may come from E protein itself to form an oligomer and release into culture medium through envelopment by vesicles. However, the total E protein contents measured in the same infected cells treated with Brefeldin A, a fungal macrolide antibiotic to disassembly the Golgi compartment and block the SP secretion, had similar levels among the wild type and prM mutants (Fig. 8J). These results indicate that the assembly, budding and secretion of SP are not only determined by the interactions among the prM and E enveloped proteins but also several post-assembly events such as budding and secretion. The newly synthesized prM and E enveloped proteins can associate to form homo- or hetero-dimeric/oligomeric complexes which can not be incorporated into an icosahedral lattice for budding into the ER lumen. The final stage of SP release in the infected cells still requires a rapid transport along the compartments of the secretory pathway. It was reported that some of the prM mutations located outside the TM region in DENV and TBE hardly affected the prM-E heterodimerization but greatly inhibited the SP secretion [26,27]. The SP release through envelop budding is driven by multimerization of the envelope proteins prM and E to create an icosahedral lattice which is composed by 60 asymmetric trimers of prM-E heterodimers [28]. Then the prM-E heterodimers, transient contacts between prM TM domains occurred during the budding process, dissociate due to the major rearrangement into the E:E dimers during the maturation process of the particles after cleavage of prM by a furin in the trans Golgi network [29]. Further investigation of SP budding and secretion particularly in late-step secretory pathways are required to understand the detailed mechanisms of flavivirus assembly, exit, and maturation.

The GXXXG motif in the prM-TM1 segment of the JEV 2195LA strain was further studied using site-specific mutagenesis to demonstrate its specificity for helix-helix interaction in the TM anchor regions. Replacing the glycine residues in the GXXXG motif with three other small amino acids (alanine, leucine and valine), supporting extensive interhelical van der Waals interactions, indicated that the two glycine residues were equally important to the formation of prM-E heterodimers (Fig. 12). This site-specific mutagenesis result is consistent with the previously reported effect of the GXXXG motif on the formation of the E1:E2 heterdimer in hepatitis C virus and Semliki Forest virus [24,30,31]. However, virus assembly depends on a replacement by leucine of the conserved glycine residues in the GXXXG motif in Semliki Forest virus [32]. Thus, the importance of the conserved glycine residues in the TM anchor regions may vary with the variations in amino acid sequence among virus species.

The topology of the TM regions of enveloped proteins of enveloped viruses has been recently revealed for the enveloped protein heterodimerization and the SP formation, but with inconclusive results [14,24,31,33,34]. In an earlier study on YFV, the alanine insertions did not affect prM-E heterodimerization but greatly impaired the SP release of YFV [13]. Alanine insertion within the center of the TMDs of E1 or E2 or in the N-terminal part of the TMD of E1 dramatically affected for 60-90% reduction of hepatitis C virus envelope glycoproteins [24]. Even the E1-E2 heterodimerization was not affected, some mutants still showed reduced HCVpp infectivity [31]. The differences among these viruses may be due to their similar topology of TM region of envelope proteins. The TM regions of prM and E proteins of flaviviruses are potentially longer than their counterpart in HCV [13,33]. Further sequence alignment analysis of the anchor regions of prM proteins of various flaviviruses, shown in Additional file 1, indicates that the LGXXLG motif is present in the TM1 region of JEV and MVE; the IGXXLG motif in KUNV, SLEV and WNV; the LAXXIG motif in DENV-1 to DENV-4; and the IAXXVG motif in the YFV. As revealed by a statistical analysis of the anchor regions of TM proteins, most of the amino acid patterns of small residues (Gly, Ala and Ser) at i and i+4 are associated with large aliphatic residues (Ile, Val and Leu) at neighboring positions (i.e. i+/-1 and i+/-2) in the TM anchor regions [35]. Sequence alignment analysis of the anchor regions of prM proteins of various flaviviruses indicates that the prM-TM1 domain of YFV contains AYLVG residues rather than the GXXXG motif. Furthermore, the connecting segment between prM-TM1 and prM-TM2 domains of JEV prM proteins has four hydrophilic residues and one charged residue (NNGQ**R**). Replacing the arginine (R) residue with alanine in the prM connected segment did not affect the prM-E heterodimerization and SP formation (data not shown). The data on SP inhibition by prM insertion mutants in the two TM segments but not the connecting segment suggest that the prM-TM1 and prM-TM2 domains may play importation roles in the formation and release of the JEV particle assembly.

## Conclusion

In this study, we characterized the involvement of JEV prM TM1 and TM2 region in the prM-E heterodimerization and the assembly of subviral particles. Furthermore, we identified a conserved GXXXG motif in the prM TM1 region of the JEV serocomplex viruses, which may play an important role in the early biosynthesis process of the virus.

## Competing interests

The authors declare that they have no competing interests.

## Authors' contributions

YJL performed the immunoprecipitation and TEM. JGP quantified the mRNA/protein expression level and analyzed the experimental data. SCW designed the experiments and wrote the manuscript. All authors read and approved the final manuscript.

## Supplementary Material

Additional file 1 **Amino acid sequence alignment analysis of TM regions of prM protein (prM130-167) from genus *Flavivirus***. The numbers of amino acid sequences of 22 flavivirus strains (NCBI and EMBL accession numbers listed) are given (the consensus amino acids symbolized by dashes). Gly142 and Gly146 of the GXXXG motif are marked with a black box. Click here for file
