# Supplementary material for: Characterization of the GXXXG motif in the first transmembrane segment of Japanese encephalitis virus precursor membrane (prM) protein
Source: J Biomed Sci. 2010 May 24;17(1):39. doi: 10.1186/1423-0127-17-39 (PMC2890656; doi:10.1186/1423-0127-17-39)
Supplement: Additional file 1 — Amino acid sequence alignment analysis of TM regions of prM protein (prM130-167) from genus Flavivirus. The numbers of amino acid sequences of 22 flavivirus strains (NCBI and EMBL accession numbers listed) are given (the consensus amino acids symbolized by dashes). Gly142 and Gly146 of the GXXXG motif are marked with a black box. [file 1423-0127-17-39-S1.DOCX]

| **A** | **JEV** | **CH2195LA** | AAF34186 | **R** | **N** | **P** | **G** | **Y** | **A** | **F** | **L** | **A** | **A** | **A** | **L** | **G** | **W** | **M** | **L** | **G** | **S** | **N** | **N** | **G** | **Q** | **R** | **V** | **V** | **F** | **T** | **I** | **L** | **L** | **L** | **L** | **V** | **A** | **P** | **A** | **Y** | **S** |
| --- | --- | --- | --- | --- | --- | --- | --- | --- | --- | --- | --- | --- | --- | --- | --- | --- | --- | --- | --- | --- | --- | --- | --- | --- | --- | --- | --- | --- | --- | --- | --- | --- | --- | --- | --- | --- | --- | --- | --- | --- | --- |
|  |  | **Beijing-1** | L48961 | **-** | **-** | **-** | **-** | **-** | **-** | **-** | **-** | **-** | **-** | **I** | **-** | **-** | **-** | **-** | **-** | **-** | **-** | **-** |  |  |  | **-** | **-** | **-** | **-** | **-** | **-** | **-** | **-** | **-** | **-** | **-** | **-** | **-** | **-** | **-** | **-** |
|  |  | **SA-14-14-2** | D90195 | **-** | **-** | **-** | **-** | **-** | **-** | **-** | **-** | **-** | **-** | **V** | **-** | **-** | **-** | **-** | **-** | **-** | **-** | **-** |  |  | **-** | **-** | **-** | **-** | **-** | **-** | **-** | **-** | **-** | **-** | **-** | **-** | **-** | **-** | **-** | **-** | **-** |
|  | **KUNV** | **MRM61C** | P14335 | **-** | **-** | **-** | **-** | **-** | **-** | **L** | **V** | **-** | **-** | **V** | **I** | **-** | **-** | **-** | **-** | **-** | **-** | **-** | **T** | **M** | **-** | **-** | **-** | **-** | **-** | **A** | **V** | **-** | **-** | **-** | **-** | **-** | **-** | **-** | **-** | **-** | **-** |
|  | **MVEV** | **MVE-1-51** | P05769 | **-** | **-** | **-** | **-** | **-** | **-** | **L** | **V** | **-** | **V** | **V** | **-** | **-** | **-** | **-** | **-** | **-** | **-** | **-** | **T** |  | **-** | **K** | **-** | **I** | **-** | **-** | **V** | **-** | **-** | **-** | **-** | **-** | **-** | **-** | **-** | **-** | **-** |
|  | **SLEV** | **MSI-7** | P09732 | **-** | **-** | **-** | **-** | **-** | **-** | **L** | **V** | **-** | **L** | **-** | **I** | **-** | **-** | **-** | **-** | **-** | **-** | **-** |  | **T** | **-** |  | **-** | **-** | **-** | **V** | **-** | **M** | **-** | **M** | **-** | **I** | **-** | **-** | **-** | **-** | **-** |
|  | **WNV** | **385-99** | M12294 | **-** | **-** | **-** | **-** | **-** | **-** | **L** | **V** | **-** | **-** | **V** | **I** | **-** | **-** | **-** | **-** | **-** | **-** | **-** | **T** | **M** | **-** |  | **-** | **-** | **-** | **A** | **-** | **-** | **-** | **-** | **-** | **-** | **-** | **-** | **-** | **-** | **-** |
| **B** | **DENV-1** | **Western Pacific** | AAN06981 | **-** | **H** | **-** | **-** | **F** | **T** | **V** | **I** | **-** | **L** | **F** | **-** | **A** | **H** | **A** | **I** | **-** | **T** | **S** | **I** | **T** | **-** | **K** | **G** | **I** | **I** | **F** | **-** | **-** | **-** | **M** | **-** | **-** | **T** | **-** | **S** | **M** | **A** |
|  |  | **AHF 82-80** | P27912 | **-** | **H** | **-** | **-** | **F** | **T** | **V** | **I** | **G** | **L** | **F** | **-** | **A** | **H** | **A** | **I** | **-** | **T** | **S** | **I** | **T** | **-** | **K** | **G** | **I** | **I** | **F** | **-** | **-** | **-** | **M** | **-** | **-** | **T** | **-** | **S** | **M** | **A** |
|  |  | **CV1636/77** | P27913 | **-** | **H** | **-** | **-** | **F** | **T** | **V** | **I** | **G** | **L** | **F** | **-** | **A** | **Y** | **T** | **I** | **-** | **T** | **S** | **I** | **T** | **-** | **K** | **G** | **I** | **I** | **F** | **-** | **-** | **-** | **M** | **-** | **-** | **T** | **-** | **S** | **M** | **A** |
|  | **DENV-2** | **PR159/S1** | P12823 | **-** | **H** | **-** | **-** | **F** | **T** | **I** | **M** | **-** | **-** | **I** | **-** | **A** | **Y** | **T** | **I** | **-** | **T** | **T** | **H** | **F** | **-** | **-** |  | **L** | **I** | **F** | **-** | **-** | **-** | **T** | **A** | **I** | **-** | **-** | **S** | **M** | **T** |
|  |  | **Jamaica** | P07564 | **-** | **H** | **-** | **-** | **F** | **T** | **I** | **M** | **-** | **-** | **I** | **-** | **A** | **Y** | **T** | **I** | **-** | **T** | **T** | **H** | **F** | **-** | **-** | **A** | **L** | **I** | **F** | **-** | **-** | **-** | **T** | **A** | **-** | **-** | **-** | **S** | **M** | **T** |
|  |  | **D2-04** | P30026 | **-** | **H** | **-** | **-** | **F** | **T** | **I** | **M** | **-** | **-** | **I** | **-** | **A** | **Y** | **T** | **I** | **-** | **T** | **T** | **H** | **F** | **-** | **-** | **A** | **L** | **I** | **F** | **-** | **-** | **Q** | **T** | **A** | **-** | **-** | **-** | **S** | **M** | **T** |
|  | **DENV-3** | **68784** | AAK74146 | **-** | **H** | **-** | **-** | **F** | **T** | **I** | **-** | **-** | **L** | **F** | **-** | **A** | **H** | **Y** | **I** | **-** | **T** | **S** | **L** | **T** | **-** | **K** | **-** | **-** | **I** | **F** | **-** | **-** | **-** | **M** | **-** | **-** | **T** | **P** | **S** | **M** | **T** |
|  |  | **Sleman/78** | AAT69740 | **-** | **H** | **-** | **-** | **F** | **T** | **I** | **-** | **-** | **L** | **F** | **-** | **A** | **H** | **Y** | **I** | **-** | **T** | **S** | **L** | **T** | **-** | **K** | **-** | **-** | **I** | **F** | **-** | **-** | **-** | **M** | **-** | **-** | **T** | **P** | **S** | **M** | **T** |
|  | **DENV-4** | **814669** | P09866 | **-** | **-** | **P** | **G** | **F** | **-** | **L** | **-** | **-** | **G** | **F** | **M** | **A** | **Y** | **-** | **I** | **-** | **Q** | **T** | **G** | **I** | **-** | **-** | **T** | **-** | **-** | **F** | **-** | **L** | **M** | **M** | **-** | **-** | **-** | **-** | **S** | **-** | **G** |
|  | **TBEV** | **Vasilchenko** | AAF82240 | **K** | **-** | **K** | **L** | **L** | **-** | **L** | **A** | **V** | **V** | **-** | **V** | **V** | **-** | **L** | **T** | **V** | **E** | **S** | **V** | **V** | **T** | **-** | **I** | **T** | **V** | **V** | **-** | **V** | **-** | **-** | **C** | **L** | **-** | **-** | **V** | **-** | **A** |
|  |  | **SOFJIN** | P07720 | **K** | **-** | **K** | **V** | **L** | **T** | **L** | **A** | **V** | **I** | **-** | **V** | **V** | **-** | **L** | **T** | **V** | **E** | **S** | **V** | **V** | **T** | **-** | **-** | **A** | **V** | **V** | **V** | **V** | **-** | **-** | **C** | **L** | **-** | **-** | **V** | **-** | **A** |
|  |  | **Neudoerfl** | P14336 | **K** | **-** | **K** | **L** | **L** | **-** | **L** | **A** | **M** | **V** | **T** | **V** | **V** | **-** | **L** | **T** | **L** | **E** | **S** | **V** | **V** | **T** | **-** | **-** | **A** | **V** | **L** | **V** | **V** | **-** | **-** | **C** | **L** | **-** | **-** | **V** | **-** | **A** |
|  | **YFV** | **17D** | P03314 | **-** | **-** | **-** | **F** | **F** | **-** | **V** | **T** | **-** | **L** | **T** | **I** | **A** | **Y** | **L** | **V** | **-** | **-** | **-** | **M** | **T** | **-** | **-** | **-** | **-** | **I** | **A** | **L** | **-** | **V** | **-** | **A** | **-** | **G** | **-** | **-** | **-** | **-** |
|  |  | **1899/81** | P29165 | **-** | **-** | **-** | **F** | **F** | **-** | **V** | **T** | **-** | **L** | **A** | **I** | **A** | **Y** | **L** | **V** | **-** | **-** | **-** | **M** | **T** | **-** | **-** | **-** | **-** | **I** | **A** | **L** | **-** | **V** | **-** | **A** | **-** | **G** | **-** | **-** | **-** | **-** |
|  |  | **PASTEUR 17D-204** | P19901 | **-** | **-** | **-** | **F** | **F** | **-** | **V** | **T** | **-** | **L** | **T** | **I** | **A** | **Y** | **L** | **V** | **-** | **-** | **-** | **M** | **T** | **-** | **-** | **-** | **-** | **I** | **A** | **L** | **-** | **V** | **-** | **A** | **-** | **G** | **-** | **-** | **-** | **-** |
